# Hypotonia, Ataxia, Developmental Delay and Tooth Enamel Defect Syndrome (HADDTS) due to a Heterozygous de Novo Missense Variant in *CTBP1* Identified via Whole Genome Sequencing

**DOI:** 10.1155/crpe/3604592

**Published:** 2025-09-08

**Authors:** Silvia Beatriz Sanchez Marco, Emily Pardington, Marie Monaghan, Robert Spaull, Ala Fadilah, Kathreena Kurian, Kayal Vijayakumar, Sarah Smithson, Anirban Majumdar

**Affiliations:** ^1^Department of Paediatric Neurology, University Hospital of Wales, Cardiff and Vale University Health Board, Cardiff, UK; ^2^Royal United Hospital, Royal United Hospitals Bath NHS Trust Foundation, Bath, UK; ^3^Department of Paediatric Neurology, Bristol Royal Hospital for Children, University Hospitals Bristol NHS Foundation Trust, Bristol, UK; ^4^Department of Neuropathology, Southmead Hospital, University Hospitals Bristol NHS Foundation Trust, Bristol, UK; ^5^Department of Clinical Genetics, St Michael's Hospital, University Hospitals Bristol NHS Foundation Trust, Bristol, UK

**Keywords:** cerebellar ataxia, CTBP1, developmental delay, enamel defects, hypotonia, whole genome sequencing

## Abstract

We describe a three-year-old girl with an unusual c-terminal binding protein 1 (*CTBP1*) gene variant. She presented with features of hypotonia, ataxia, developmental delay and tooth enamel defect syndrome (HADDTS), following numerous chest infections, poor weight gain and delayed motor development during the early years. After many years of genetic testing where no diagnosis was found, whole genome sequencing (WGS) identified a missense variant in the *CTBP1* gene (NM_001012614.1): c.991C > T p.(Arg331Trp). We present some of the brain MRI (cerebellar atrophy) and muscle biopsy features (central nuclei/cores) characteristic of this condition. The underlying mechanisms have not yet been elucidated. Although the clinical features make this condition recognisable, we are aware that in the small community of patients with this condition, the time to diagnosis may be exceptionally long. WGS has allowed us to accelerate this process. We are hopeful that earlier identification will bring better care for the affected children and allow the genetic implications to be discussed with their families.

## 1. Introduction

Developmental delay associated with hypotonia can give rise to a large number of potential differential diagnoses in paediatrics. This can lead to many, often invasive, neurometabolic investigations in the hope of finding the aetiology. New genetic testing techniques, such as whole exome sequencing (WES) and whole genome sequencing (WGS), have revolutionised diagnosis, with the identification of specific gene variants causing disease. The 100,000-genome project was WGS based, with the main beneficiaries being those children and their families with rare diseases, offering new possibilities for diagnosis and the potential for future treatments [1]. We present the case of a female patient presenting with hypotonia and developmental delay with a long diagnostic journey concluded by access to this project.

## 2. Case Report

### 2.1. Case Description and Clinical Features

The patient was born at full term by uncomplicated vaginal delivery with a birth weight of 2.74 kg (5^th^ centile). She is the second child of nonconsanguineous parents. There was no family history of note. During the neonatal period, she presented with recurrent chest infections. Weight gain was also suboptimal during this period. There were delayed gross motor milestones as well as delayed language development, more so of expressive than receptive skills. Crawling was achieved, though parents noted no further motor development by the age of 3 years. Fine motor skills were unaffected.

She was 3 years old at the time of initial neurology assessment. She was noted to have a distinctive facial phenotype with fine blonde hair, a pointed chin and ears with blue sclerae. There was also syndactyly of the 2^nd^ and 3^rd^ toes, and the patient remained symmetrically small with a head circumference below the 0.4^th^ centile. She also presented hypotonia, muscle weakness, upbeat nystagmus, oculomotor apraxia, global developmental delay and ataxia.

During this period, she continued to have poor weight gain with unsafe swallowing; therefore, she was started on gastrostomy feeds, which helped to improve weight gain and energy levels, allowing her to begin crawling again. By the age of 4 years, the patient was able to walk short distances with full support of a walker due to balance problems and during this time was found to have in toeing and mild scoliosis of the spine, most evident when she was tired.

Management for recurrent respiratory infections was ongoing throughout this presentation. A cough assist device was initiated for daily use as well as prophylactic amoxicillin for respiratory infections and saline nebulisers. Sleep studies showed mild nocturnal hypoventilation not requiring any treatment.

Following initial motor improvements after the gastrostomy, there was then a period of regression of motor skills. Having had success at walking with a frame, at 5 years old, the patient lost this ability and required a powered wheelchair for mobility. During this time, she was noted to have developed spasticity of the lower limbs, which required treatment with botulinum toxin for symptomatic relief.

### 2.2. Summary of Investigations

As part of an initial workup, blood tests including a full blood count and electrolytes were performed revealing a low urea and creatinine, reflective of reduced muscle bulk. Neurometabolic testing included white cell enzymes, lysosomal enzymes, very long-chain fatty acids and sugar chromatography. Genetic studies for mitochondrial DNA variants were negative.

Brain MRI at age 4 years old showed mild cerebellar folia hypoplasia with, otherwise, normal intracranial appearances.

**Figure 1:**
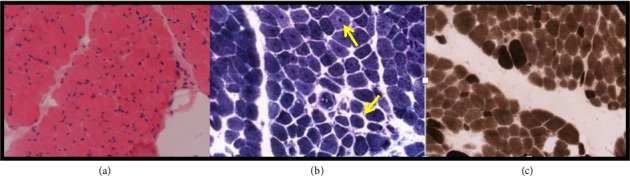
Muscle biopsy taken from quadriceps muscle. (a) Haematoxylin and Eosin (H&E) stain shows significant variation in fibres size with atrophic myofibers. (b) NADH stain: central nuclei in some fibres (yellow arrows). (c) ATPase stain with marked type 1 muscle fibres predominance. Findings are suggestive of congenital centronuclear/myotubular myopathy.

Nerve conduction studies were normal. Electromyography (EMG) showed signs of myopathy. Following these tests, a muscle biopsy was taken at age of 3 years old, which showed significant variation in fibres size with atrophic myofibers and central nuclei in some fibres suggestive of congenital centronuclear/myotubular myopathy (Figure 1).

### 2.3. Genetic Findings

Further genetic testing included array CGH, chromosome 15q methylation, investigation of *EPG5* (VICI syndrome) and standard gene panels for myopathy genes *MTM1*, *BIN1* and *DNM2*; these were all normal. The patient was referred to the 100,000 Genomes Project.

Sequencing identified a significant *de novo* heterozygous missense variant [2] in the gene encoding c-terminal binding protein 1 (CTBP1): *CTBP1* (NM_001012614.2): c.991C > T p.(Arg331Trp) as a rare cause of hypotonia, developmental delay and tooth enamel defect syndrome (HADDTS) [2, 3]. This variant has been classified as a pathogenic variant using ACMG/AMP guidelines [3, 4].

## 3. Discussion

We present a child with a *CTBP1* variant causing HADDTS. Due to the condition being so rare, these patients are often undiagnosed. This highlights the ongoing importance of WGS in improving knowledge of rare conditions, including HADDTS and offering early diagnosis for these patients [5]. Long term, this may also help develop potential treatments for this condition [6, 7]. Diagnosis is also crucial for families, as it allows them to connect and form a community of support with others in their situation, and often, as in this case, to demonstrate a low risk of having a further affected child in the family [8].

The *CTBP1* gene maps to chromosome 4p16 and encodes a transcriptional regulator, which interacts with chromatin-modifying enzymes to modulate gene expression in a number of cellular pathways [9]. It is thought to function mainly through repression through recruitment of histone-modifying enzymes and methyltranderases [2, 10].

*CTBP1* has multiple domains that mediate protein–protein interactions including PDZ, NAD(H), Arg–Arg–Thr (RRT) and Pro–Leu–Asp–Leu–Ser (PLDLS) binding cleft domains—the locus of the Arg331Trp variant [2]. Through the PLDLS domain, CTBP1 plays a critical role as a scaffolding protein for transcription factors and chromatin-modifying enzymes usually liked to neurodevelopmental disorders, autism and congenital heart disease [2]. The mutation at this point may cause changes in chromatin modification and thereby affect gene expression in multiple tissues, hence the multifaceted phenotype of HADDTS [2].

The *CTBP1* variant has rarely been reported in the literature, although there have been animal studies described looking at the role of the CTBP1 channel itself [2, 3, 11].

Comparing the phenotype of the patient to the literature available (Table 1), she presented very similarly [11–13]. The clinical cases described presented with developmental delay, facial dysmorphism, such as frontal bossing, deep-set eyes, long faces, tooth enamel defects in the form of enamel hypoplasia with significant balance problems, poor weight gain and had evidence of cerebellar atrophy on MRI. More recently, Zhang et al. described a patient with distinctive facial features, developmental delay but without hypotonia, ataxic gait or tooth enamel defect noticed [14].

Muscle biopsies of these patients also showed a similar myopathic process with fibre diameter variability with atrophy, dystrophy features, endomysial fibrosis, internal/central nuclei and clumps [10, 15].

The pathogenic variant identified in this case, *CTBP1* (NM_001012614.2): c.991C > T p. (Arg331Trp), has been reported in the literature in 14 cases [2, 11, 16, 17]. Two other pathogenic variants, a *CTBP1* missense variant (NM_001328.3: c.1024C > T; p.Arg342Trp) with a similar clinical phenotype and a *CTBP1* variant (NM_001328.2: c.371C > T (p.Ser124Phe) without hypotonia, ataxic gait or tooth enamel defect have also been reported [14, 15].

The patient described in this article is an example of the challenges clinicians face in the management and diagnosis of complex patients. It illustrates the clinical phenotype and progression of the condition from birth and the different investigation results, including muscle biopsy findings and genetic results, which will help clinicians regarding early diagnosis of this rare syndrome. As highlighted in this manuscript, the progress in the genetic testing field offers an essential tool in the diagnosis and management of these patients.

## 4. Conclusion

HADDTS is a rare syndrome associated with pathogenic variants at the *CTBP1* gene. Further reports are needed to better understand and identify this frequent underdiagnosed condition. Genetic testing plays an essential role in the diagnosis and promotion of research regarding further therapeutical options for these patients.

## Figures and Tables

**Table 1:** Phenotypic spectrum of HADDTS described in the literature [1, 2, 6, 12–16].

Case	Age at description (years)	Gender	Global developmental delay	Enamel defects	Hypotonia	Ataxia	Difficulty with weight gain	Distinctive features	Others	Brain MRI	ENG/EMG	Muscle biopsy	References
I	12	Female	+	+	+	+	+	N/A	Dysarthria	Normal	N/A	Dystrophic changes/fibre variability size and shape	Beck et al. (2016)
II	8	Male	+	+	+	+	+	N/A	Dysarthria	Cerebellar volume loss/vermian atrophy	N/A	Perivascular inflammation	Beck et al. (2016)
III	20	Male	+	+	+	+	+	Frontal bossing/deep set eyes	Wolf–Parkinson–White syndrome	Normal	N/A	Varied muscle fibre size/chronic dystrophy or myopathy changes	Beck et al. (2016)
IV	9	Female	+	+	+	+	+	Retrognathia/highly arched palate	Dysarthria/mild nystagmus	Diminished volume of cerebellar superior vermis	N/A	Inflammatory infiltrates and occasional necrotic fibres	Beck et al. (2016)
V	16	Female	+	+	+	+	+	Deep set eyes/thin fingers	Contractures wrists and elbows/scoliosis	Mild cerebellar and brainstem atrophy	N/A	Variation in muscle fibre size, occasional internal nuclei and denervation atrophy	Sommervile et al. (2017)
VI	20	Male	+	N/A	+	+	N/A	N/A	N/A	N/A	Fibrillation activity myopathic process	N/A	Beck et al. (2019)
VII	22	Female	+	+	+	+	N/A	N/A	Contractures wrists and elbows/horizontal nystagmus	Progressive cerebellar and cerebral volume loss	Bilateral ulnar mononeuropathies	N/A	Beck et al. (2019)
VIII	6	Male	+	+	+	+	N/A	N/A	Dysarthria	Normal	N/A	N/A	Beck et al. (2019)
IX	6	Male	+	+	+	+	N/A	N/A	N/A	Cerebellar atrophy	Mild to moderate chronic nonirritable myopathy	N/A	Beck et al. (2019)
X	10	Male	+	+	+	+	N/A	N/A	N/A	Enlargement of the cisterna magna and hypoplasia of the inferior vermis	N/A	N/A	Beck et al. (2019)
XI	5	Male	+	N/A	+	+	N/A	N/A	Dysarthria	Walker cyst, superior cerebellar vermis volume loss	N/A	N/A	Beck et al. (2019)
XII	11	Male	+	+	+	+	N/A	N/A	Dysarthria and dysmetria	Vermian atrophy/hemispheric cerebellar volume loss with associated volume loss of the superior and inferior cerebellar peduncles and the pons	N/A	N/A	Beck et al. (2019)
XIII	7	Male	+	+	+	+	+	Long face	Contractures upper and lower limbs scoliosis dysarthria	Prominent cerebellar foliae	Low-amplitude motor unit action potential of 0.3–0.5 mV indicative of myopathic pattern	Features of muscular dystrophy	Bhatia et al. (2020)
XIV	25	Male	+	+	−	+/−	Normal weight	Frontal bossing/deep-set eyes	Dysarthria, nystagmus and one myoclonic seizure	N/A	N/A	N/A	Khamirani et al. (2021)
XV	15	Male	+	+	+	+	+	Claw-feet and hammer toes	Intellectual disability	Cerebellar hemispheric and vermian atrophy	Myopathic pattern	Dystrophic features with endomysial-fibrosis, fibre-size variability, necrotic/degenerative vacuolar myopathy, sarcoplasmic/myofibrillar and vacuolar mitochondriopathy.	Khadim et al. (2023)
XVI	3	Female	+	−	−	−	+	Microcephaly, a visible beard, synophrys, low-set ears. Single transverse palmar crease, a short fifth finger	Congenital dislocation of the radial head, pectus excavatum. Mild tricuspid valve regurgitation myopia	Normal	N/A	N/A	Zhang et al. (2024)
XVII	3	Female	+	+	+	+	+	Fine blonde hair, a pointed chin and ears with blue sclerae. There was also syndactyly of the 2nd and 3rd toes	Upbeat nystagmus	Mild cerebellar folia hypoplasia	Suggestive of myopathy.	Variation in fibres size with atrophic myofibers and central nuclei suggestive of congenital centronuclear/myotubular myopathy	^∗^Sanchez Marco et al. (2025)

^∗^N/A: Nonapplicable (data not collected).

## Data Availability

The data that support the findings of this study are available from the corresponding author upon reasonable request.
